# Multifaceted Assessment of Functional Outcomes in Survivors of First-time Stroke

**DOI:** 10.1001/jamanetworkopen.2022.33094

**Published:** 2022-09-23

**Authors:** Seyoung Shin, Yaesuel Lee, Won Hyuk Chang, Min Kyun Sohn, Jongmin Lee, Deog Young Kim, Yong-Il Shin, Gyung-Jae Oh, Yang-Soo Lee, Min Cheol Joo, So Young Lee, Min-Keun Song, Junhee Han, Jeonghoon Ahn, Yun-Hee Kim

**Affiliations:** 1 Department of Physical and Rehabilitation Medicine, Center for Prevention and Rehabilitation, Heart Vascular Stroke Institute, Samsung Medical Center, Sungkyunkwan University School of Medicine, Seoul, Republic of Korea; 2 Department of Rehabilitation Medicine, College of Medicine, Chungnam National University, Daejeon, Republic of Korea; 3 Department of Rehabilitation Medicine, Konkuk University School of Medicine, Seoul, Republic of Korea; 4 Department and Research Institute of Rehabilitation Medicine, Yonsei University College of Medicine, Seoul, Republic of Korea; 5 Department of Rehabilitation Medicine, Pusan National University School of Medicine, Pusan National University Yangsan Hospital, Yangsan, Republic of Korea; 6 Department of Preventive Medicine, Wonkwang University, School of Medicine, Iksan, Republic of Korea; 7 Department of Rehabilitation Medicine, Kyungpook National University School of Medicine, Kyungpook National University Hospital, Daegu, Republic of Korea; 8 Department of Rehabilitation Medicine, Wonkwang University School of Medicine, Iksan, Republic of Korea; 9 Department of Rehabilitation Medicine, Jeju National University Hospital, Jeju National University School of Medicine, Jeju City, Republic of Korea; 10 Department of Physical and Rehabilitation Medicine, Chonnam National University Medical School, Gwangju, Republic of Korea; 11 Department of Statistics, Hallym University, Chuncheon, Republic of Korea; 12 Department of Health Convergence, Ewha Womans University, Seoul, Republic of Korea; 13 Department of Health Science and Technology, Department of Medical Devices Management and Research, Department of Digital Healthcare, Samsung Advanced Institute for Health Sciences and Technology, Sungkyunkwan University, Seoul, Republic of Korea

## Abstract

**Importance:**

Because stroke causes diverse functional deficits, understanding the long-term recovery pattern of each functional domain may inform prognosis and therapeutic strategies.

**Objective:**

To observe long-term changes in functional status and residual disability in survivors of first-time stroke.

**Design, Setting, and Participants:**

This cohort study was an interim analysis of the Korean Stroke Cohort for Functioning and Rehabilitation. Between August 2012 and May 2015, 7858 of 10 636 screened patients with first-time strokes from 9 district hospitals in Korea provided informed consent to participate. Data were analyzed from September 2021 through February 2022.

**Exposure:**

First-time stroke.

**Main Outcomes and Measures:**

Study data include multifaceted face-to-face functional assessments obtained at 8 to 9 points until 60 months after stroke onset. The Korean Mini-Mental State Examination (K-MMSE), Fugl-Meyer Assessment, Functional Ambulatory Category, American Speech-Language-Hearing Association National Outcome Measurement System Swallowing Scale, and Short Korean version of the Frenchay Aphasia Screening Test were performed from 7 days to 60 months after stroke. The Korean Modified Barthel Index was measured from 3 months to 60 months after stroke.

**Results:**

A total of 4443 patients (2649 men [59.62%]; mean [SD] age 62.13 [12.43] years) who underwent repeated functional assessments for 60 months after stroke (3508 patients with ischemic and 935 patients with hemorrhagic stroke) were included. Overall, functions plateaued between 12 and 18 months after stroke and declined after 30 months; for example, mean (SD) K-MMSE improved from 7 days (22.89 [7.89]) to 12 months (26.03 [5.48]) (*P* < .001), plateaued until 36 months (26.03 [5.84]), and decreased to 48 months (26.02 [5.82]) (*P* < .001). Interaction associations were found between time after stroke and age, stroke severity, and stroke type in functional assessment outcomes. For example, mean (SE) FMA for ages 65 years or younger vs older than 65 years was 81.64 (0.63) vs 80.69 (0.68) at 7 days and 91.28 (0.47) vs 88.46 (0.58) at 6 months (*P* for interaction < .001), and for IS vs HS, it was 84.46 (0.47) vs 69.02 (1.24) at 7 days and 91.20 (0.38) vs 85.51 (0.98) at 6 months (*P* for interaction < .001). Mean (SE) FMA was 94.39 (0.21) at 7 days and 97.57 (0.14) at 6 months for mild stroke, 44.69 (1.18) at 7 days and 70.43 (1.21) at 6 months for moderate stroke, and 13.22 (0.99) at 7 days and 48.07 (2.62) at 6 months for severe stroke (*P* for interaction < .001). Factors associated with activities of daily living independence at 60 months included older age (β per 1-year increase = −0.35; standard error [SE], 0.03; *P* < .001), male sex (β = 2.12; SE, 0.73; *P* = .004), and hemorrhagic stroke type (β vs ischemic stroke = 2.35; SE, 0.81; *P* = .004).

**Conclusions and Relevance:**

This study found that long-term recovery patterns in multifaceted functional domains differed from one another and varied by patient age, stroke severity, and stroke type. Understanding the diversity of long-term functional recovery patterns and factors associated with these outcomes in survivors of stroke may help clinicians develop strategies for effective stroke care and rehabilitation.

## Key Points

**Question:**

How do long-term functional recovery patterns after stroke differ by functional domain and what factors are associated with these outcomes?

**Findings:**

In this cohort study of 4443 patients with strokes, early recovery curve, time to plateau, and long-term decline pattern differed by functional domain, age, initial stroke severity, and stroke type. Clinical and functional factors, including age, sex, and stroke type, were associated with activities of daily living performance at 60 months.

**Meaning:**

These findings may help clinicians develop better strategies for effective early stroke care and rehabilitation.

## Teaser

This cohort study investigates changes in functional status up to 60 months after stroke in patients experiencing first-time stroke in Korea.

## Introduction

An aging population and decreased stroke mortality have led to increased global burden of the disease as more patients are surviving with functional deficits after stroke.^1,2^ A thorough understanding of long-term functional outcomes after stroke is important for setting suitable management plans and correctly informing patients and their families about prognoses.^3^ Previous studies^4,5,6^ have reported stroke outcome as a level of independence or mortality among survivors of stroke. Many stroke cohort studies have shown that initial stroke severity and baseline clinical characteristics are associated with long-term mortality,^7^ functional independence,^8,9^ and cognitive impairment.^10^

Overall disability and mortality are the most important stroke outcomes; however, stroke is associated with diverse functional impairments that vary by lesion location or size. A patient with aphasia could receive good scores on the Barthel Index or modified Rankin Score (mRS) despite experiencing a major functional deficit in communication after a stroke.^11^ Therefore, long-term outcomes in different functional domains need to be understood separately to establish adequate stroke management strategies in accordance with individual patient needs. Moreover, it would be worthwhile to examine whether functional recovery patterns differ by clinical characteristics already known to be associated with stroke prognosis, such as age, stroke type, or stroke severity.^3,4,12,13^ Previous stroke outcome studies^5,9,14^ examined some early clinical factors associated with 5-year poststroke functional outcomes, although most of them were conducted with relatively few patients.

In this study, we examined long-term recovery patterns in diverse functional domains and factors associated with outcomes among patients with first-time stroke. We analyzed data from a single large cohort in which repeated face-to-face assessments of motor skills, cognition, ambulation, language, and swallowing function were assessed, which was an advantage over previous studies.

## Methods

The Korean Stroke Cohort for Functioning and Rehabilitation (KOSCO) is a cohort study of patients with acute first-time strokes who were admitted to representative hospitals in 9 distinct areas of Korea.^15^ Written informed consent was obtained from all patients prior to inclusion in the study, and the study protocol was approved by the institutional review board (IRB) of each participating hospital. The original study’s IRB approvals extend until December 2031 because it is an ongoing cohort study. This study was a retrospective analysis of KOSCO data obtained through the natural course of disease (stroke recovery), did not apply additional intervention or assessment, and so did not require additional IRB approval. Patients provided informed consent to participate in this follow-up cohort study. This study followed the Strengthening the Reporting of Observational Studies in Epidemiology (STROBE) reporting guideline for observational studies.

The KOSCO study collected multifaceted, serial, face-to-face functional assessment data for patients with strokes up to 60 months after their first stroke onset. The detailed rationale and protocol for KOSCO were described in a previous article.^15^

### Study Participants

Between August 2012 and May 2015, 7858 of 10 636 screened patients with first-time strokes from 9 district hospitals in Korea provided informed consent to participate in this follow-up study. Of them, 6253 patients (79.57%) experienced ischemic stroke (IS) and 1605 patients (20.43%) experienced hemorrhagic stroke (HS). During the first 60 months after stroke onset, 1674 patients (21.3%), including 1416 patients with IS and 258 patients with HS, died and 1741 patients (22.2%), including 1329 patients with IS and 412 patients with HS, were lost to follow-up. Therefore, 4443 patients with stroke, including 3508 patients with IS (79.0%) and 935 patients with HS (21.0%) who continued with follow-up assessment until 60 months after onset were included in this analysis (eFigure in the Supplement).

### Functional Assessments

Multifaceted functional assessments were performed 7 days and 3, 6, 12, 18, 24, 30, 36, 48, and 60 months after stroke onset using the Korean Mini-Mental State Examination (K-MMSE)^16^ for cognitive function (score range, 0-30, with higher scores indicating better performance), Fugl-Meyer Assessment (FMA)^17^ for motor impairment (range, 0-100, with higher scores representing better motor function), Functional Ambulatory Category (FAC)^18^ for ambulatory function (score range, 0-5, with higher scores representing better ambulatory function), American Speech-Language-Hearing Association National Outcome Measurement System Swallowing Scale (ASHA-NOMS)^19^ for swallowing function (score range 1-7, with higher scores indicating better swallowing function), and Short Korean version of the Frenchay Aphasia Screening Test (Short K-FAST)^20^ for language function (score range, 0-20, with language function considered to be good with higher scores). Functional independence in the activities of daily living (ADL) was measured at 3, 6, 12, 18, 24, 30, 36, 48, and 60 months after stroke using the Korean modified Barthel Index (K-MBI; range 0-100, with higher K-MBI scores indicating better ADL independence).^21^ Licensed occupational and physical therapists completed a designated training program and then participated as assessors in face-to-face functional assessments. To maintain the quality of the assessments and inter-rater reliability, assessors underwent repeated training every 3 months during the study. All functional assessments were considered as continuous variables with intervals of 1 unit in this analysis.

### Patient Demographic and Clinical Characteristics

Data on clinical characteristics, including comorbidities, demographic characteristics (age, sex, body mass index [BMI; calculated as weight in kilograms divided by height in meters squared], marital status, smoking and alcohol history, and education level), and prestroke functional levels, were documented by members of the hospital staff during acute treatment. Comorbidities were assessed using the combined condition- and age-related score in the Charlson Comorbidity Index (CCAS).^22^ Prestroke functional level was assessed using mRS.^23^ The following risk factors were recorded during admission to the hospital: hypertension, diabetes, coronary disease, atrial fibrillation, and hyperlipidemia (eMethods in the Supplement). Age, education year, CCAS, and mRS (range, 0-6) were considered as continuous variables with intervals of 1 unit or 1 year in this analysis.

Stroke severity was assessed using the National Institute of Health Stroke Scale (NIHSS; score range, 0-42)^24^ for IS and Glasgow Coma Scale (score range, 3-15) for HS. Scores in medical records at the time of arrival to the emergency department were used. NIHSS scores at 7 days after onset were also assessed in both stroke types as unified baseline stroke severity score. A previous study^25^ found that NIHSS was a reliable tool for clinical monitoring in patients with intracranial hemorrhage. According to NIHSS score at 7 days, patients were grouped into 3 categories of mild (NIHSS, 0-4), moderate (NIHSS, 5-15), and severe (NIHSS, 16-42) stroke.^26^

During hospitalization, information was recorded about serious medical complications, including pneumonia, urinary tract infection, thromboembolic disease, ventilatory insufficiency, and pressure sores. Duration of hospitalization and inpatient rehabilitation status were also recorded.

### Statistical Analysis

Descriptive statistics were used for baseline patient characteristics and multifaceted functional outcomes for 60 months after the first stroke occurrence. Numerical variables are summarized as means and SDs, and categorical variables are summarized as numbers and percentages. To examine differences between stroke types, each variable was analyzed using independent *t* or χ^2^ testing. Paired *t* testing with Bonferroni correction was performed to evaluate the time to plateau in each functional domain, separately and for subsets grouped by age, 7-day stroke severity (NIHSS) score, and stroke type. The level of significance was set as 2-sided *P* < .05. Differences in functional recovery over time by subgroup were analyzed using generalized estimating equations, a statistical technique for comparing longitudinal data containing repeated observations. For this analysis, adjustment factors were considered. Covariates were baseline characteristics that differed significantly (*P* < .05) between subgroups. To delineate factors associated with functional outcomes of patients with stroke after 60 months, multivariable linear regression analyses were performed with factors that were statistically significant (*P* < .05) in univariable linear analyses (eMethods in the Supplement). Missing data at each time were excluded from analysis. Analyses were performed using R statistical software version 4.0.3 (R Project for Statistical Computing) and SPSS statistical software version 27.0 (IBM). Data were analyzed from September 2021 through February 2022.

## Results

### Patient Characteristics

The Table shows demographic and clinical characteristics of participants. Among 4443 patients (mean [SD] age, 62.13 [12.43] years; 2649 men [59.62%]), there were 3508 patients with IS (mean [SD] age, 63.47 [12.00] years; 2192 [62.49%] men) and 935 patients with HS (mean [SD] age, 57.09 [12.73] years; 457 [48.88%] men). Patients with HS had significantly more comorbidities than patients with IS (hypertension: 384 patients [41.07%] vs 1849 patients [52.71%]; diabetes: 79 patients [8.45%] vs 823 patients [23.46%]; coronary heart disease 26 patients [2.78%] vs 208 patients [5.93%]; atrial fibrillation: 15 patients [1.60%] vs 263 patients [7.50%]; hyperlipidemia: 38 patients [4.06%] vs 372 patients [10.60%]) and higher mean [SD] CCAS (4.66 [1.61] vs 5.20 [1.58]). However, mean (SD) premorbid mRS was higher in patients with HS compared with patients with IS (0.87 [1.59] vs 0.61 [1.17]). Additionally, the prevalence of pneumonia (46 patients [4.92%] vs 47 patients [1.34%]; *P* < .001), urinary tract infection (45 patients [4.81%] vs 49 patients [1.40%]; *P* < .001), ventilatory insufficiency (9 patients [0/96%] vs 12 patients [0.34%]; *P* < .001), and pressure sores during first hospitalization (7 patients [0.75%] vs 4 patients [0.11%]; *P* < .001) was significantly higher in patients with HS than patients with IS. The prevalence of thromboembolic disease did not differ significantly between patients with HS and those with IS (10 patients [1.07%] vs 44 patients [1.25%]; *P* = .77).

**Table.** Demographic and Clinical Characteristics of Participants

Characteristic	Participants, No. (%)			*P* value^a^
	Total (N = 4443)	Ischemic stroke (n = 3508)	Hemorrhagic stroke (n = 935)	
Age, mean (SD), y	62.13 (12.43)	63.47 (12.00)	57.09 (12.73)	<.001
Sex				
Men	2649 (59.62)	2192 (62.49)	457 (48.88)	<.001
Women	1794 (40.38)	1316 (37.51)	478 (51.12)	
BMI, mean (SD)	23.89 (3.24)	23.99 (3.24)	23.51 (3.19)	<.001
Smoking, current	1220 (27.46)	1006 (28.68)	214 (22.89)	<.001
Alcohol use, current	1849 (41.62)	1444 (41.16)	405 (43.32)	.25
Education, mean (SD), y	10.11 (4.70)	9.93 (4.73)	10.77 (4.50)	<.001
Married	3423 (77.04)	2703 (77.05)	720 (77.01)	.99
Medical history				
Hypertension	2233 (50.26)	1849 (52.71)	384 (41.07)	<.001
Diabetes	902 (20.30)	823 (23.46)	79 (8.45)	<.001
Coronary heart disease	234 (5.27)	208 (5.93)	26 (2.78)	<.001
Atrial fibrillation	278 (6.26)	263 (7.50)	15 (1.60)	<.001
Hyperlipidemia	410 (9.23)	372 (10.60)	38 (4.06)	<.001
CCAS, mean (SD)	5.09 (1.60)	5.20 (1.58)	4.66 (1.61)	<.001
Premorbid mRS score, mean (SD)	0.67 (1.27)	0.61 (1.17)	0.87 (1.59)	<.001
Initial severity, mean (SD)				
Initial NIHSS score	4.17 (4.68)	4.17 (4.68)	NA	NA
Initial GCS	13.07 (3.07)	NA	13.07 (3.07)	NA
Complications during hospitalization				
Pneumonia	93 (2.09)	47 (1.34)	46 (4.92)	<.001
Urinary tract infection	94 (2.12)	49 (1.40)	45 (4.81)	<.001
Thromboembolic disease	54 (1.22)	44 (1.25)	10 (1.07)	.77
Ventilatory insufficiency	21 (0.47)	12 (0.34)	9 (0.96)	.03
Pressure sore	11 (0.25)	4 (0.11)	7 (0.75)	.002
Duration of hospitalization, mean (SD), d	17.85 (23.76)	13.75 (19.78)	33.25 (30.26)	<.001
Inpatient rehabilitation	856 (19.27)	565 (16.11)	292 (31.12)	<.001

Abbreviations: BMI, body mass index (calculated as weight in kilograms divided by height in meters squared); CCAS, combined condition- and age-related score in the Charlson Comorbidity Index; GCS, Glasgow Coma Scale; mRS, modified Rankin Scale; NA, not applicable; NIHSS, National Institutes of Health Stroke Scale.

^a^ Independent *t* test of ischemic and hemorrhagic stroke types.

### Multifaceted Functional Outcomes Over 60 Months After First-time Stroke

**Figure 1.**
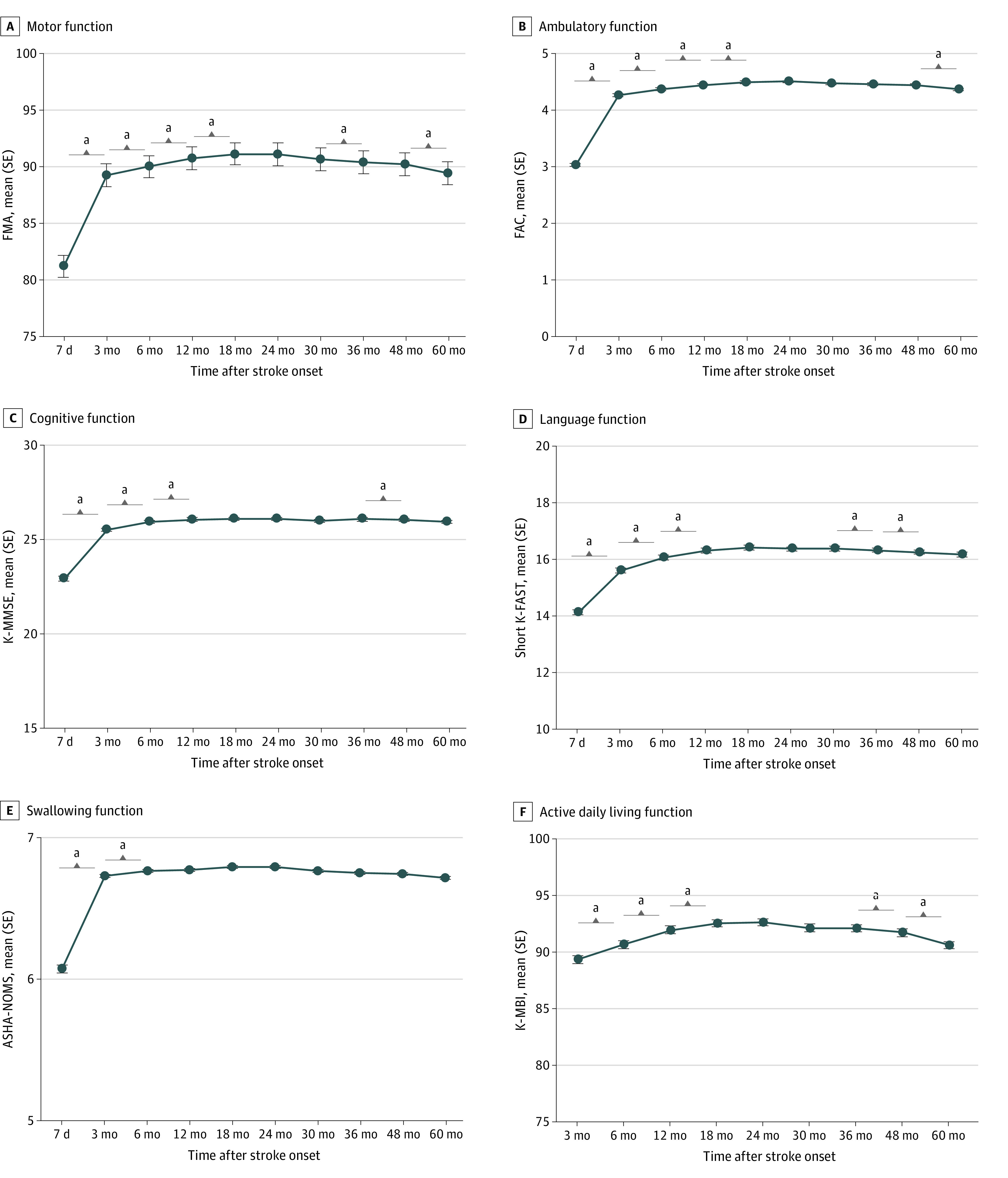
Multifaceted Functional Recovery Patterns of All Participants Multifaceted functional recovery patterns of 4443 participants during 60 months after stroke onset for motor, ambulatory, cognitive, language, swallowing, and activities of daily living functions. The x-axis represents time after stroke, and the y-axis represents functional assessment scores. ASHA-NOMS indicates American Speech-Language-Hearing Association National Outcome Measurement System Swallowing Scale; dots, mean scores; FAC, Functional Ambulatory Category; FMA, Fugl-Meyer Assessment; K-MBI, Korean modified Barthel Index; K-MMSE, Korean Mini-Mental State Examination; Short K-FAST, Short Korean version of the Frenchay Aphasia Screening Test; whiskers, standard errors. ^a^*P* < .05 compared with the previous time point after Bonferroni correction.

Figure 1 and eTable 1 in the Supplement show multifaceted functional outcomes of patients with first-time stroke assessed repeatedly from 7 days to 60 months after stroke onset. Mean (SD) FMA score showed significant improvement from 7 days (81.21 [30.73]) to 18 months (91.14 [21.29]) after stroke onset (for 7 days to 6 months, *P* < .001; for 12 months to 18 months, *P* = .001) and then plateaued until 30 months (90.66 [21.99]). It decreased significantly after that, to 89.44 (23.07) (*P* = .004). Mean (SD) FAC increased significantly from 7 days (3.03 [1.88]) to 18 months (4.49 [1.19]) after stroke (for all segments, *P* < .001) and then plateaued until 48 months (4.44 [1.26]). It then decreased until 60 months (4.36 [1.34]) after stroke (*P* < .001). Mean (SD) K-MMSE score improved significantly from 7 days (22.89 [7.89]) to 12 months (26.03 [5.48]) after stroke (for all segments, *P* < .001) and then plateaued until 36 months (26.03 [5.84]). It decreased from 36 to 48 months (26.02 [5.82]) after stroke (*P* < .001). Mean Short K-FAST score improved from 7 days (14.18 [5.71]) to 12 months (16.36 [4.66]) after stroke onset (for all segments, *P* < .001) and then plateaued until 30 months (16.44 [4.74]). It decreased significantly after 30 months, to 16.31 (4.84) at 48 months (for 36 to 48 months, *P* = .005; for 48 to 60 months, *P* < .001). Mean (SD) ASHA-NOMS score improved significantly from 7 days (6.08 [1.79]) to 6 months (6.78 [0.76]) after stroke (*P* < .001) and then plateaued until 60 months (6.73 [0.84]). Mean (SD) K-MBI score improved significantly from 3 months (89.33 [21.68]) to 18 months (92.56 [19.02]) after stroke onset (for all segments, *P* < .001) and then plateaued until 36 months (92.12 [20.41]). From 36 to 60 months (90.61 [22.85]), it decreased significantly (for all segments, *P* < .001).

**Figure 2.**
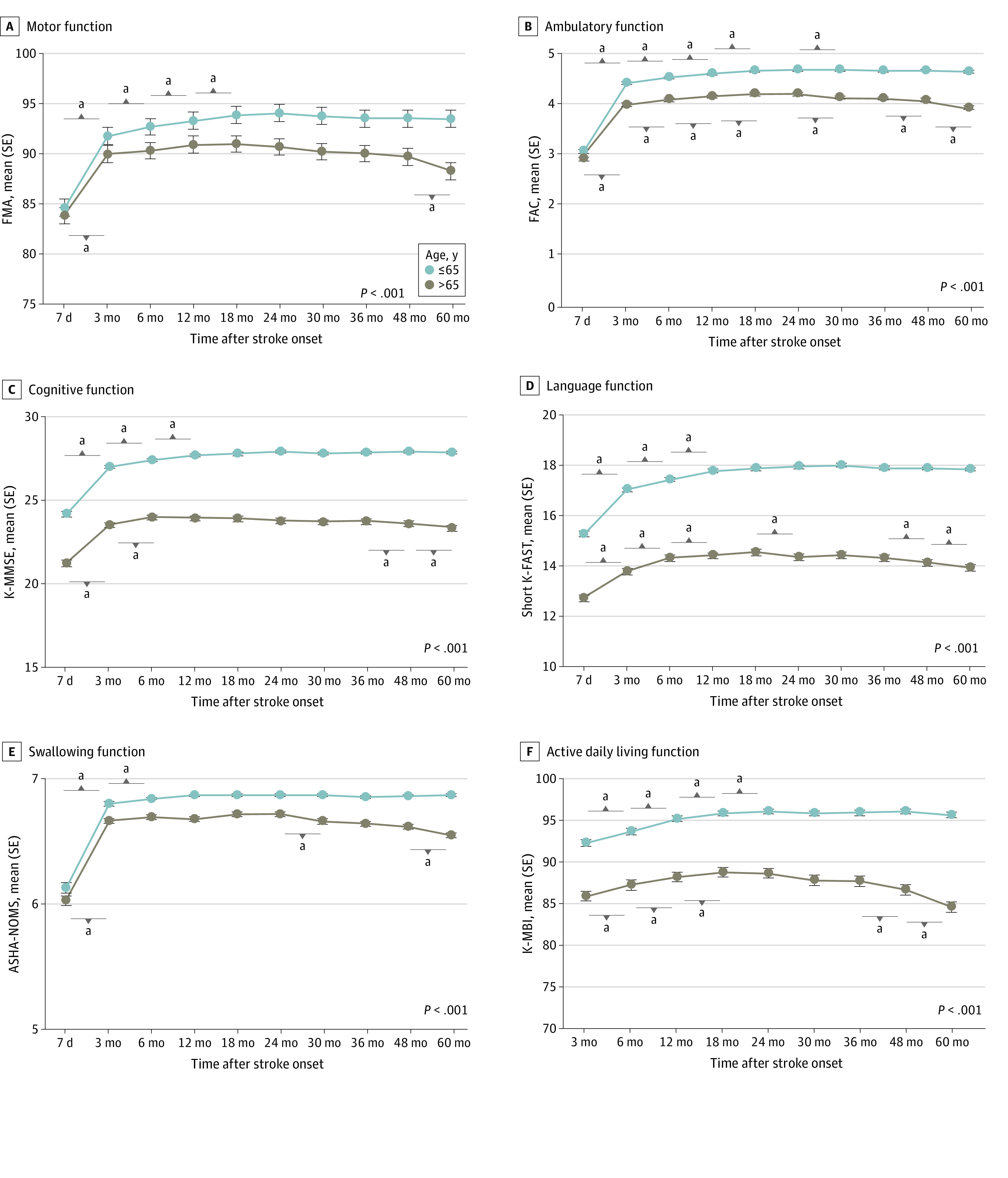
Multifaceted Functional Recovery Patterns by Age Subgroup analysis of functional recovery patterns by age during 60 months after stroke onset for motor, ambulatory, cognitive, language, swallowing, and activities of daily living functions. The x-axis represents time after stroke, and the y-axis demonstrates functional assessments. *P* < .001 by generalized estimating equation analysis between patients ages 65 years or younger and those older than 65 years is indicated on plots. ASHA-NOMS indicates American Speech-Language-Hearing Association National Outcome Measurement System Swallowing Scale; dots, mean scores; FAC, Functional Ambulatory Category; FMA, Fugl-Meyer Assessment; K-MBI, Korean modified Barthel Index; K-MMSE, Korean Mini-Mental State Examination; Short K-FAST, Short Korean version of the Frenchay Aphasia Screening Test; whiskers, standard errors. ^a^*P* < .05 compared with the previous time point after Bonferroni correction.

Figure 2 shows multifaceted functional recovery patterns in patients with first-time stroke by age group (≤65 years vs >65 years). There was an interaction association between time and age group in FMA, FAC, K-MMSE, Short K-FAST, ASHA-NOMS, and K-MBI scores after adjustment (eTable 2 in the Supplement). For example, mean (SE) FMA for ages 65 years or younger vs older than 65 years was 81.64 (0.63) vs 80.69 (0.68) at 7 days and 91.28 (0.47) vs 88.46 (0.58) at 6 months (*P* for interaction < .001). Therefore, multifaceted functional recovery patterns differed by age. In young and older groups, every functional domain showed significant improvements during the subacute phase; for example, mean (SE) FMA at 3 months improved to 90.20 (0.46) for patients aged 65 years or younger (*P* < .001) and 88.04 (0.59) for patients older than 65 years (*P* < .001). However, the mean score in each domain remained lower in the older group than in the younger group, and those discrepancies increased over time. The younger group showed continuous improvement until 12 to 24 months for most functions and did not experience further deterioration, whereas the older group demonstrated functional declines in the chronic phase, which differed by function (ie, 48 months to 60 months in motor function; 36 months to 48 months in ambulatory, cognitive, language, and ADL functions; and 24-35 months and 48-60 months for swallowing function).

**Figure 3.**
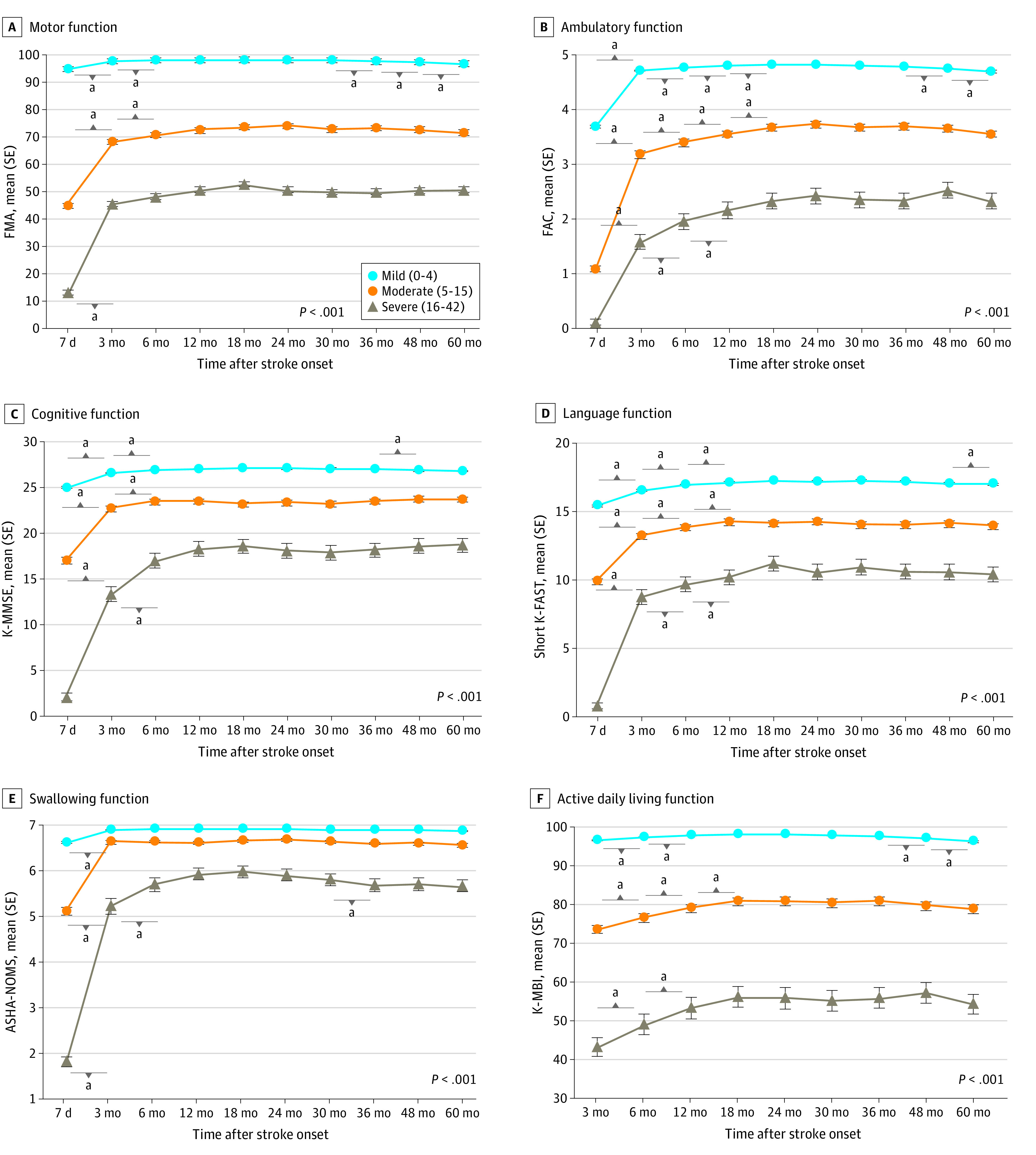
Multifaceted Functional Recovery Patterns by Stroke Severity Subgroup analysis of functional recovery patterns by 7-day stroke severity during 60 months after stroke onset for motor, ambulatory, cognitive, language, swallowing, and activities of daily living functions. The x-axis represents time after stroke, and the y-axis demonstrates functional assessments. *P* < .001 by generalized estimating equation analysis among subgroups of initial stroke severity is indicated on plots. ASHA-NOMS indicates American Speech-Language-Hearing Association National Outcome Measurement System Swallowing Scale; dots, mean scores; FAC, Functional Ambulatory Category; FMA, Fugl-Meyer Assessment; K-MBI, Korean modified Barthel Index; K-MMSE, Korean Mini-Mental State Examination; Short K-FAST, Short Korean version of the Frenchay Aphasia Screening Test; whiskers, standard errors. ^a^*P* < .05 compared with the previous time point after Bonferroni correction.

Figure 3 shows multifaceted functional recovery patterns by stroke severity (mild, moderate, and severe) as measured by the NIHSS at 7 days. The generalized estimating equation analysis showed an interaction association between time and stroke severity in all 6 functional domains after adjustment (eTable 2 in the Supplement). For example, mean (SE) FMA was 94.39 (0.21) at 7 days and 97.57 (0.14) at 6 months for mild stroke, 44.69 (1.18) at 7 days and 70.43 (1.21) at 6 months for moderate stroke, and 13.22 (0.99) at 7 days and 48.07 (2.62) at 6 months for severe stroke (*P* for interaction < .001). Results indicated that multifaceted functional recovery patterns differed by initial stroke severity. Moderate and severe groups showed rapid improvement in the subacute phase (for example, mean [SE] FMA at 3 months improved to 67.99 [1.24] for moderate stroke [*P* < .001] and 45.46 [2.61] for severe stroke [*P* < .001]), and all 3 groups showed the most significant improvement during the first 12 months after stroke.

**Figure 4.**
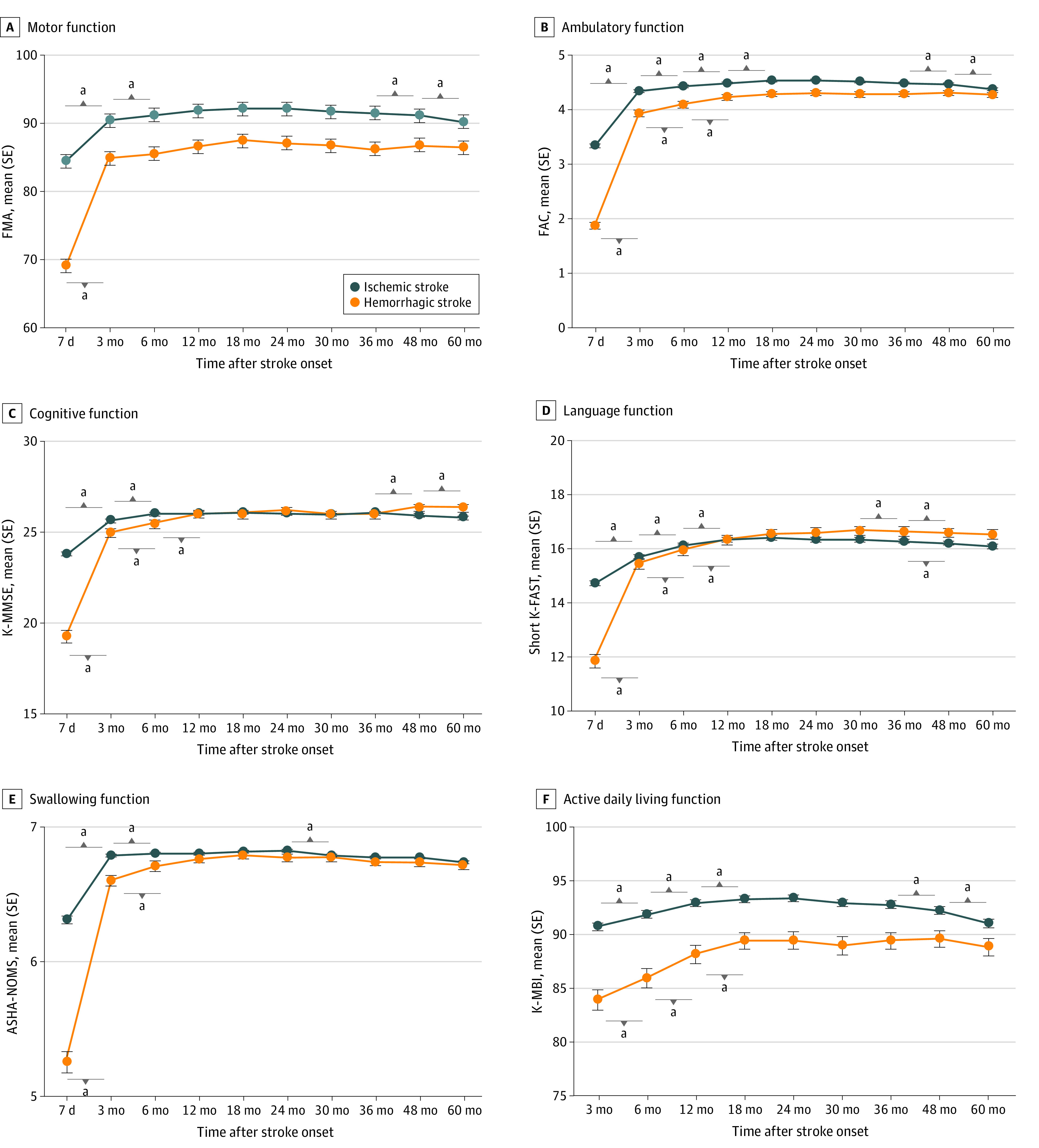
Multifaceted Functional Recovery Patterns by Stroke Type Subgroup analysis of functional recovery patterns by stroke type, ischemic or hemorrhagic, during 60 months after stroke onset for motor, ambulatory, cognitive, language, swallowing, and activities of daily living functions. The x-axis represents time after stroke, and the y-axis demonstrates each functional assessment. *P* < .001 by generalized estimating equation analysis between stroke types, ischemic or hemorrhagic, is indicated on plots. ASHA-NOMS indicates American Speech-Language-Hearing Association National Outcome Measurement System Swallowing Scale; dots, mean scores; FAC, Functional Ambulatory Category; FMA, Fugl-Meyer Assessment; K-MBI, Korean modified Barthel Index; K-MMSE, Korean Mini-Mental State Examination; Short K-FAST, Short Korean version of the Frenchay Aphasia Screening Test; whiskers, standard errors. ^a^*P* < .05 compared with the previous time point after Bonferroni correction.

Figure 4 shows multifaceted functional outcomes by stroke type. Results showed an interaction association between time and stroke type in all functional domains after adjustment (eTable 2 in the Supplement). For example, mean (SE) FMA for IS vs HS was 84.46 (0.47) vs 69.02 (1.24) at 7 days and 91.20 (0.38) vs 85.51 (0.98) at 6 months (*P* for interaction < .001). Multifaceted functional recovery patterns differed by stroke type; patients with HS tended to show more drastic improvement during the subacute phase in every functional domain compared with patients with IS. For example, mean (SE) FMA improved at 3 months to 84.85 (0.99) for HS (*P* < .001) and 90.41 (0.40) for IS (*P* < .001). However, patients with IS showed significant functional decreases in the chronic phase after 36 months, whereas patients with HS remained relatively stable over time.

### Factors Associated With Long-term Functional Outcomes in Survivors of First-time Stroke

Results of our univariable regression analyses are summarized in eTable 3 in the Supplement; eTable 4 in the Supplement shows results of multivariable linear regression analyses using significant factors extracted from univariable analysis. HS, more years of education, and higher CCAS, FMA, FAC, and K-MMSE scores at 7 days after stroke onset were positive factors associated with motor outcome at 60 months. Older age, history of diabetes, and higher 7-day NIHSS score were negative factors associated with long-term motor outcome. For ambulatory function, multivariable regression analysis showed that HS, male sex, more years of education, and higher CCAS, FMA, K-MMSE, and FAC scores at 7 days after stroke were positively associated with function, but older age, diabetes history, and higher 7-day NIHSS score were negatively associated with outcome. For the cognitive outcome, HS, male sex, higher BMI, and higher CCAS, K-MMSE, and short K-FAST scores at 7 days were positively associated with the outcome, but older age, atrial fibrillation, and higher 7-day NIHSS score were negatively associated with cognitive outcome. For language function, HS, male sex, more years of education, and higher CCAS, FMA, K-MMSE, and short K-FAST scores at 7 days after stroke onset were positively associated with the outcome. Older age, history of atrial fibrillation, and higher 7-day NIHSS score were negatively associated with language outcome. For swallowing function, higher CCAS, K-MMSE, and ASHA-NOMS scores at 7 days after stroke onset were positively associated with the outcome, but older age, history of atrial fibrillation, higher 7-day NIHSS score, and lower short K-FAST score at 7 days were negatively associated with the outcome. For ADL independence, HS (β = 2.35; SE, 0.81; *P* = .004), male sex (β = 2.12; SE, 0.73; *P* = .004), more years of education (β = 0.21; SE, 0.08; *P* = .006), and higher CCAS (β = 1.13; SE, 0.22; *P* < .001), FMA (β = 0.10; SE, 0.02; *P* < .001), K-MMSE (β = 0.38; SE, 0.08; *P* < .001), and FAC (β = 0.60; SE, 0.23; *P* = .009) scores at 7 days after stroke onset were positively associated with the outcome, but older age (β = −0.35; SE, 0.03; *P* < .001), diabetes (β = −1.82; SE, 0.75; *P* = .02), and higher 7-day NIHSS score (β = −1.07; SE, 0.14; *P* < .001) were negatively associated with the outcome.

## Discussion

In this cohort study, we found differences among long-term outcomes and recovery patterns for various functional domains after stroke. Functional scores showed significant improvement in most domains until 12 or 18 months after stroke onset and then plateaued. From 30 months after onset, functional levels decreased in most domains. When considering covariates, recovery patterns differed significantly by age, initial stroke severity, and stroke type.

Some reports have suggested that ADL and motor functions plateau after 6 months^2,27^; however, our results demonstrated that recovery continued for more than 1 year in all functional domains and until 18 months in motor, ambulatory, and ADL functions. These outcomes may be associated with improvements in structural stroke care and comprehensive rehabilitation services in recent years. Functional recovery occurred rapidly in acute and subacute phases of stroke, especially in younger patients, those with moderate or severe stroke severity, and patients with HS. Those phases are a time when patients should receive as much restorative therapy as needed to obtain maximal recovery.^2^ In the chronic stage of stroke, on the other hand, functional decline occurred beginning 30 months after onset, which suggests that maintenance care strategies are of utmost importance at that time. A previous report^10^ found that late recovery was associated with reduced mortality and health care cost in patients with stroke, suggesting that stroke care should be extended to provide rehabilitation services needed by individual patients even in the chronic phase. The primary strength of our study is that it was a large single cohort with repeated multifaceted functional assessments for 60 months. Face-to-face assessment by qualified assessors of patients who experienced strokes enabled us to accurately evaluate various functions.

Age is an important factor associated with functional outcomes after stroke. The older group in our study had lower scores than the younger group in all functional domains at every time point. Older patients with stroke had noticeable functional recovery during the subacute phase, but most functions declined from 18 to 60 months after stroke. This finding complemented results of a previous study^12^ that found functional decline between 6 and 30 months after stroke in patients older than age 70 years. These findings emphasize that older patients with stroke should be monitored closely for possible functional deterioration in the chronic stage.

Functional recovery patterns in our study also differed by stroke severity; moderate and severe groups had rapid recovery during the first 6 to 12 months after stroke. By stroke type, the HS group had higher severity at onset but more rapid restoration of function in the first 12 months after stroke than the IS group. This finding is congruent with a previous study^13^ that reported that patients with HS were likely to have noticeable functional improvement compared with those with IS. The later decline in the IS group in our study may be associated with the older mean age of patients with IS compared with those with HS.

Several studies^8,9,10,28^ have used the mRS or Barthel Index to build evidence about stroke recovery and associated factors during a few months to several years after stroke. Most previous cohort studies have suggested that age, prestroke disability, initial stroke severity, education level, diabetes, depression, and other underlying diseases are common factors associated with dependency or disability after stroke.^5,8,9,14,29^ Although independence is the most important and meaningful outcome for survivors of stroke, the condition can be associated with various residual impairments that cannot be fully assessed by a single measure.^2,11^ Our multivariable regression analysis revealed that nonmodifiable factors of age, comorbidities, and initial stroke severity were associated with all functional domains. Contrary to previous study findings, premorbid disability was not associated with outcomes 60 months after stroke. This may suggest that the nature of the current stroke and subsequent treatment were more important for long-term functional outcomes than premorbid functional levels. We also found that functional status at 7 days after stroke was associated with outcomes of other functional domains after 60 months. Higher 7-day K-MMSE score was associated with good functional outcomes in all 6 functional domains, including motor, ambulatory, swallowing, language, and ADL performance. A previous neuroimaging study^30^ proposed a possible interaction between motor recovery and cognitive function; this cross-interaction of functional domains during recovery should be further investigated.

### Limitations

This study has several limitations. The main limitation of our study is that we included only patients with a complete data set for most variables, which may have influenced functional outcomes through the healthy worker effect. Additionally, the KOSCO study enrolled participants during 2012 to 2015. Therefore, the results did not reflect the characteristics and outcomes of patients who experienced stroke at different times. In order to overcome this limitation, the KOSCO study enrolled the second stroke cohort in 2020. In future studies, we will be able to compare stroke outcomes by time period.

## Conclusions

Stroke produces long-term residual disabilities across diverse aspects of human functioning. This cohort study may make a useful contribution to the literature by revealing distinct functional recovery patterns and identifying factors associated with multifaceted functional outcomes in long-term prospective follow-up data from patients who experienced strokes. Further research is needed to propose individualized therapeutic strategies for patients who experienced strokes with regard to specific functional deficits.

## Notes

Supplement. **eFigure.** Flowchart of Participants
eMethods.
**eTable 1.** Multifaceted Functional Outcomes at Each Assessment Point in Survivors of First-Time Stroke **eTable 2.** Generalized Estimating Equations for Functional Outcomes by Subgroup **eTable 3.** Univariable Regression Analyses for Factors Potentially Associated With Functional Outcomes at 60 mo After First-Time Stroke **eTable 4.** Multivariable Regression Analyses for Factors Potentially Associated With Functional Outcomes at 60 mo After First-Time Stroke
