# Supplementary material for: Multifaceted Assessment of Functional Outcomes in Survivors of First-time Stroke
Source: JAMA Netw Open. 2022 Sep 23;5(9):e2233094. doi: 10.1001/jamanetworkopen.2022.33094 (PMC9508656; doi:10.1001/jamanetworkopen.2022.33094)
Supplement: Supplement. — eFigure. Flowchart of Participants eMethods. eTable 1. Multifaceted Functional Outcomes at Each Assessment Point in Survivors of First-Time Stroke eTable 2. Generalized Estimating Equations for Functional Outcomes by Subgroup eTable 3. Univariable Regression Analyses for Factors Potentially Associated With Functional Outcomes at 60 mo After First-Time Stroke eTable 4. Multivariable Regression Analyses for Factors Potentially Associated With Functional Outcomes at 60 mo After First-Time Stroke [file jamanetwopen-e2233094-s001.pdf]

Supplemental Online Content

Shin S, Lee Y, Chang WH, et al. Multifaceted assessment of functional outcomes in survivors of first-time stroke. *JAMA Netw Open*. 2022;5(9):e2233094. doi:10.1001/jamanetworkopen.2022.33094

eFigure. Flowchart of Participants

eMethods.

eTable 1. Multifaceted Functional Outcomes at Each Assessment Point in Survivors of First-Time Stroke

eTable 2. Generalized Estimating Equations for Functional Outcomes by Subgroup

eTable 3. Univariable Regression Analyses for Factors Potentially Associated With Functional Outcomes at 60 mo After First-Time Stroke

eTable 4. Multivariable Regression Analyses for Factors Potentially Associated With Functional Outcomes at 60 mo After First-Time Stroke

This supplemental material has been provided by the authors to give readers additional information about their work.

© 2022 Shin S et al. *JAMA Network Open.*

eFigure. Flowchart of Participants

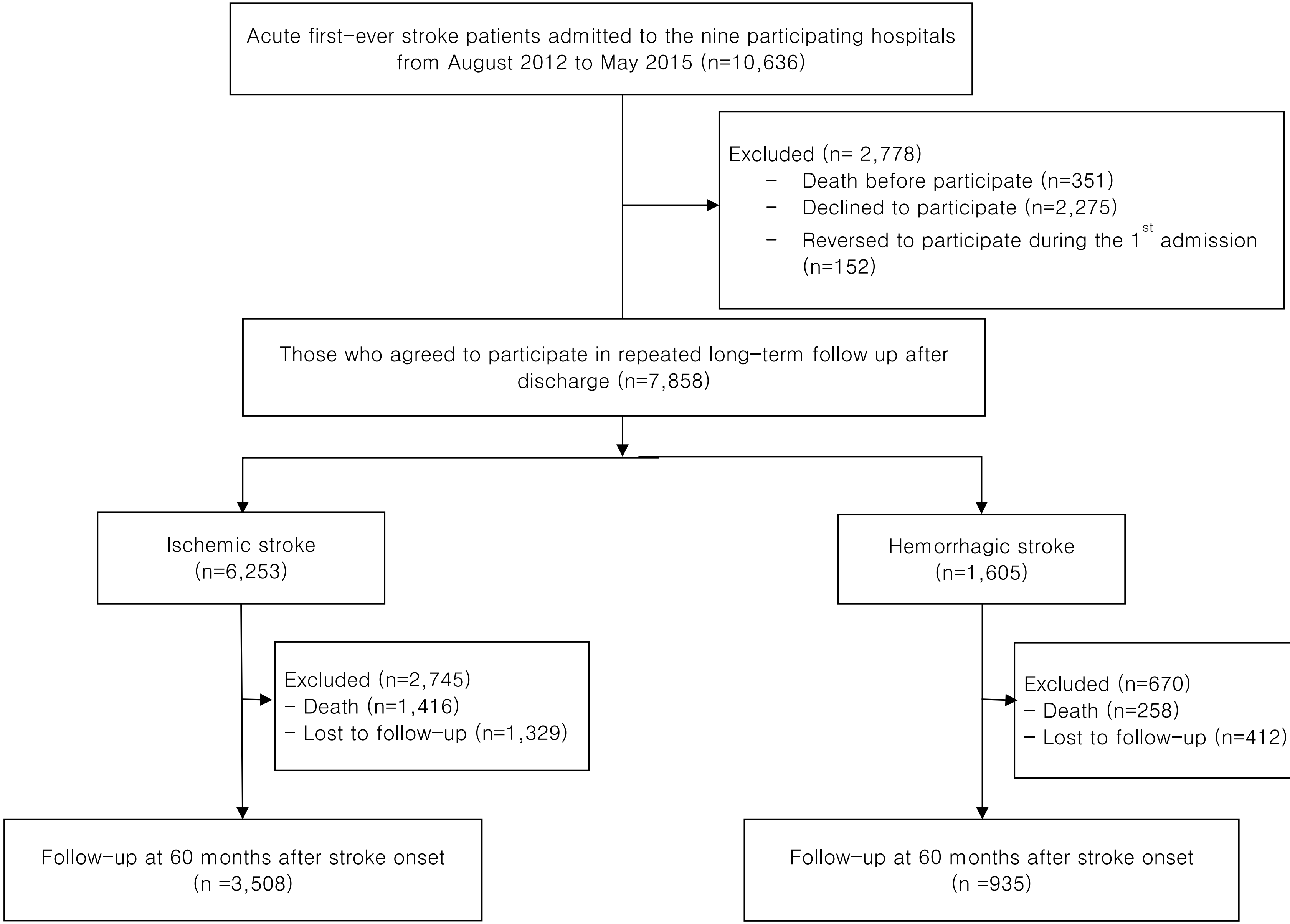

© 2022 Shin S et al. *JAMA Network Open.*

eMethods

Definitions of risk factors

Hypertension: systolic blood pressure > 160 mm Hg, diastolic blood pressure > 90 mm Hg, or history of hypertension or medical treatment
Diabetes mellitus (DM): elevated blood glucose level >126 mg/d or history of DM or medical treatment
Coronary disease: documented by standard electrocardiogram (ECG) or coronary imaging study or history of coronary disease or medical treatment
Atrial fibrillation: documented by standard ECG, long-term ECG, or history of atrial fibrillation or medical treatment
Hyperlipidemia: elevated low-density lipoprotein cholesterol level >160 mg/dL, elevated total cholesterol level > 240 mg/dL, or history of hyperlipidemia or medical treatment.

Covariate variables used in generalized estimating equations (GEEs)

The GEE analyses in this study were performed using the following covariate variables:

a. Comparison according to age: stroke type, sex, body mass index (BMI), smoking and alcohol use, education level, marital status, hypertension, diabetes mellitus (DM), coronary heart disease, atrial fibrillation, hyperlipidemia, combined condition- and age-related score in the Charlson comorbidity index (CCAS), premorbid modified Rankin Scale (mRS), 7-day National Institutes of Health Stroke Scale (NIHSS) score, duration of first hospitalization, and inpatient rehabilitation.
b. Comparison according to stroke severity: stroke type, sex, BMI, education level, marital status, DM, hyperlipidemia, premorbid mRS, pneumonia and UTI during hospitalization, duration of first hospitalization, and inpatient rehabilitation.
c. Comparison according to stroke type: age, sex, BMI, smoking, education level, hypertension, DM, coronary heart disease, atrial fibrillation, hyperlipidemia, CCAS, premorbid mRS, pneumonia and UTI during hospitalization, duration of first hospitalization, and inpatient rehabilitation.

© 2022 Shin S et al. *JAMA Network Open.*

eTable 1. Multifaceted Functional Outcomes at Each Assessment Point in Survivors of First-Time Stroke

	7 days	3 months	6 months	12 months	18 months	24 months	30 months	36 months	48 months	60 months
**FMA**										
Total stroke	81.21±30.73	89.23±23.47*	90.01±22.60[a]	90.75±21.59[a]	91.14±21.29[a]	91.11±21.48	90.66±21.99	90.39±22.00[a]	90.24±22.22	89.44±23.07[a]
Ischemic stroke	84.46±27.59	90.41±21.83[a]	91.20±20.88[a]	91.85±20.10	92.11±20.03	92.15±19.97	91.69±20.67	91.50±20.53	91.15±21.02[a]	90.24±22.08[a]
Hemorrhagic stroke	69.02±37.99	84.85±28.33[a]	85.51±27.70	86.55±26.12	87.45±25.22	87.08±26.16	86.72±26.12	86.21±26.45	86.81±26.02	86.44±26.23
**FAC**										
Total stroke	3.03±1.88	4.26±1.39[a]	4.36±1.31[a]	4.44±1.23[a]	4.49±1.19[a]	4.50±1.18	4.47±1.23	4.45±1.24	4.44±1.26	4.36±1.34[a]
Ischemic stroke	3.34±1.74	4.34±1.29[a]	4.43±1.22[a]	4.49±1.16[a]	4.54±1.12[a]	4.55±1.13	4.51±1.18	4.49±1.20	4.47±1.23[a]	4.38±1.32[a]
Hemorrhagic stroke	1.86±1.95	3.93±1.67[a]	4.10±1.59[a]	4.23±1.46[a]	4.29±1.38	4.31±1.36	4.28±1.41	4.29±1.38	4.31±1.37	4.27±1.42
**K-MMSE**										
Total stroke	22.89±7.89	25.48±5.71[a]	25.89±5.54[a]	26.03±5.48[a]	26.06±5.64	26.05±5.78	25.97±5.85	26.03±5.84	26.02±5.82[a]	25.90±5.96
Ischemic stroke	23.80±7.02	25.62±5.48[a]	26.01±5.23[a]	26.04±5.34	26.08±5.52	26.03±5.71	25.97±5.72	26.05±5.66	25.94±5.84[a]	25.79±5.98[a]
Hemorrhagic stroke	19.21±9.90	24.95±6.49[a]	25.45±6.58[a]	25.99±5.97[a]	25.97±6.10	26.14±6.04	25.95±6.35	25.95±6.48	26.33±5.73	26.33±5.85
**Short K-FAST**										
Total stroke	14.18±5.71	15.66±4.94[a]	16.12±4.71[a]	16.36±4.66[a]	16.47±4.67	16.42±4.78	16.44±4.74	16.38±4.79[a]	16.31±4.84[a]	16.22±4.96
Ischemic stroke	14.74±5.18	15.71±4.83[a]	16.16±4.58[a]	16.37±4.55[a]	16.44±4.63	16.37±4.72	16.38±4.70	16.31±4.74[a]	16.23±4.84[a]	16.13±4.96
Hemorrhagic stroke	11.87±7.05	15.47±5.33[a]	15.96±5.20[a]	16.35±5.06[a]	16.57±4.83	16.63±5.00	16.68±4.90	16.66±4.98	16.61±4.83[a]	16.56±4.96
**AHSA-NOMS**										
Total stroke	6.08±1.79	6.74± .87[a]	6.78± .76[a]	6.79± .68	6.81± .66	6.81± .63	6.78± .71	6.76± .73	6.76± .74	6.73± .84
Ischemic stroke	6.31±1.55	6.78± .74[a]	6.80± .70[a]	6.80± .61	6.81± .62	6.82± .56	6.78± .68[a]	6.77± .67	6.76± .71	6.73± .82
Hemorrhagic stroke	5.25±2.31	6.59±1.23[a]	6.70±1.02[a]	6.76± .88	6.79± .78	6.76± .84	6.77± .81	6.73± .90	6.73± .84	6.71± .91
**K-MBI**										
Total stroke	..	89.33±21.68	90.67±20.91[a]	91.97±19.74[a]	92.56±19.02[a]	92.64±19.50	92.14±20.39	92.12±20.41	91.72±21.06[a]	90.61±22.85[a]
Ischemic stroke	..	90.77±19.64	91.91±19.02[a]	92.94±18.30[a]	93.36±17.96[a]	93.45±18.48	92.96±19.40	92.82±19.61	92.27±20.47[a]	91.07±22.36[a]
Hemorrhagic stroke	..	84.00±27.34	85.99±26.34[a]	88.22±24.11[a]	89.48±22.35[a]	89.50±22.78	89.01±23.54	89.46±23.00	89.63±23.09	88.88±24.55

[a]*P* < .05 compared with previous assessment point after Bonferroni correction.

Abbreviations: FMA, Fugl-Meyer Assessment; FAC, Functional Ambulatory Category; K-MMSE, Korean Mini-Mental State Examination; Short K-FAST, Short Korean version of the Frenchay Aphasia Screening Test; AHSA-NOMS, American Speech-Language-Hearing Association National Outcome Measurement System Swallowing Scale; K-MBI, Korean modified Barthel Index;

© 2022 Shin S et al. *JAMA Network Open.*

eTable 2. Generalized Estimating Equations for Functional Outcomes by Subgroup

	**Adjusted P Value**		
	**Age [a]**	**Stroke severity [b]**	**Stroke type [c]**
FMA	<.001	<.001	<.001
FAC	<.001	<.001	<.001
K-MMSE	<.001	<.001	<.001
Short K-FAST	<.001	<.001	<.001
ASHA-NOMS	<.001	<.001	<.001
K-MBI	<.001	<.001	<.001

[a] Older than or younger than 65 years.

[b] Stroke severity: mild;NIHSS 0–4, moderate; NIHSS 5–15, severe; NIHSS 16–42.

[c] Ischemic or hemorrhagic.
Abbreviations: FMA, Fugl-Meyer Assessment; FAC, Functional Ambulatory Category; K-MMSE, Korean Mini-Mental State Examination; Short K-FAST, Short Korean version of the Frenchay Aphasia Screening Test; AHSA-NOMS, American Speech-Language-Hearing Association National Outcome Measurement System Swallowing Scale; K-MBI, Korean modified Barthel Index.

© 2022 Shin S et al. *JAMA Network Open.*

eTable 3. Univariable Regression Analyses for Factors Potentially Associated With Functional Outcomes at 60 mo After First-Time Stroke

	**FMA**		**FAC**		**K-MMSE**		**Short K-FAST**		**AHSA-NOMS**		**K-MBI**	
	**ß (SE)**	***P* Value**	**ß (SE)**	***P* Value**	**ß (SE)**	***P* Value**	**ß (SE)**	***P* Value**	**ß (SE)**	***P* Value**	**ß (SE)**	***P* Value**
Stroke type, hemorrhagic	-3.78 (0.86)	<.001	-0.11 (0.05)	.03	0.54 (0.23)	.02	0.43 (0.19)	.02	-0.02 (0.03)	.46	-2.18 (0.85)	.0106
Age	-0.29 (0.03)	<.001	-0.03 (0.002)	<.001	-0.2 (0.01)	<.001	-0.18 (0.01)	<.001	-0.02 (0.001)	<.001	-0.5 (0.03)	<.001
Sex, male	4.32 (0.71)	<.001	0.44 (0.04)	<.001	2.56 (0.19)	<.001	2.19 (0.15)	<.001	0.17 (0.03)	<.001	6.51 (0.7)	<.001
Body mass index	0.34 (0.11)	.002	0.03 (0.006)	<.001	0.17 (0.03)	<.001	0.15 (0.02)	<.001	0.02 (0.004)	<.001	0.56 (0.11)	<.001
Smoking, current	3.13 (0.79)	<.001	0.34 (0.05)	<.001	1.71 (0.21)	<.001	1.49 (0.17)	<.001	0.15 (0.03)	<.001	5.15 (0.78)	<.001
Alcohol, current	4.34 (0.71)	<.001	0.39 (0.04)	<.001	2.06 (0.19)	<.001	1.83 (0.15)	<.001	0.19 (0.03)	<.001	6.33 (0.7)	<.001
Education years	0.83 (0.08)	<.001	0.07 (0.004)	<.001	0.54 (0.02)	<.001	0.52 (0.01)	<.001	0.03 (0.003)	<.001	1.08 (0.07)	<.001
Medical history												
Hypertension	-2.16 (0.71)	.002	-0.22 (0.04)	<.001	-1.35 (0.19)	<.001	-1.05 (0.16)	<.001	-0.08 (0.03)	.002	-3.04 (0.71)	<.001
Diabetes mellitus	-2.23 (0.87)	.01	-0.2 (0.05)	.0001	-0.64 (0.23)	.005	-0.66 (0.19)	.0006	-0.04 (0.03)	.18	-2.16 (0.87)	.0128
Coronary heart disease	-0.16 (1.59)	.92	-0.04 (0.09)	.704	-0.68 (0.42)	.11	-0.16 (0.35)	.64	-0.07 (0.06)	.24	-0.08 (1.57)	.961
Atrial fibrillation	-6.07 (1.45)	<.001	-0.39 (0.08)	<.001	-2.41 (0.39)	<.001	-1.63 (0.32)	<.001	-0.23 (0.05)	<.001	-6.38 (1.43)	<.001
Hyperlipidemia	0.35 (1.21)	.772	-0.05 (0.07)	.51	0.49 (0.32)	.13	0.56 (0.27)	.04	-0.07 (0.04)	.15	0.04 (1.21)	.975
CCAS	-0.92 (0.22)	<.001	-0.08 (0.01)	<.001	-0.67 (0.06)	<.001	-0.6 (0.05)	<.001	-0.03 (0.01)	.0004	-1.07 (0.22)	<.001
Premorbid mRS	-1.09 (0.28)	<.001	-0.07 (0.02)	<.001	-0.1 (0.07)	.17	-0.1 (0.06)	.1	-0.01 (0.01)	.14	-1.26 (0.27)	<.001
7-day NIHSS	-2.36 (0.05)	<.001	-0.12 (0.003)	<.001	-0.39 (0.02)	<.001	-0.34 (0.01)	<.001	-0.05 (0.002)	<.001	-1.99 (0.05)	<.001
Functional levels at day 7												
FMA	0.47 (0.01)	<.001	0.02 (0.001)	<.001	0.06 (0.003)	<.001	0.05 (0.002)	<.001	0.01 (0.0004)	<.001	0.34 (0.01)	<.001
K-MMSE	1.13 (0.04)	<.001	0.07 (0.002)	<.001	0.39 (0.01)	<.001	0.33 (0.01)	<.001	0.03 (0.001)	<.001	1.14 (0.04)	<.001
FAC	5.7 (0.17)	<.001	0.29 (0.01)	<.001	0.84 (0.05)	<.001	0.74 (0.04)	<.001	0.11 (0.01)	<.001	4.66 (0.17)	<.001
AHSA-NOMS	5.64 (0.18)	<.001	0.28 (0.01)	<.001	0.91 (0.05)	<.001	0.8 (0.04)	<.001	0.14 (0.01)	<.001	4.94 (0.18)	<.001
Short K-FAST	1.31 (0.05)	<.001	0.08 (0.003)	<.001	0.49 (0.01)	<.001	0.44 (0.01)	<.001	0.03 (0.002)	<.001	1.35 (0.05)	<.001

Abbreviations: SE, Standard Error; FMA, Fugl-Meyer Assessment; FAC, Functional Ambulatory Category; K-MMSE, Korean Mini-Mental State Examination; Short K-FAST, Short Korean version of the Frenchay Aphasia Screening Test; AHSA-NOMS, American Speech-Language-Hearing Association National Outcome Measurement System Swallowing Scale; K-MBI, Korean modified Barthel Index; CCAS, combined condition- and age-related score in the Charlson comorbidity index; mRS, modified Rankin scale; NIHSS, National Institutes of Health Stroke Scale.

© 2022 Shin S et al. *JAMA Network Open.*

eTable 4. Multivariable Regression Analyses for Factors Potentially Associated With Functional Outcomes at 60 mo After First-Time Stroke

	FMA		FAC		K-MMSE		Short K-FAST		AHSA-NOMS		K-MBI	
	**ß (SE)**	***P* Value**	**ß (SE)**	***P* Value**	**ß (SE)**	***P* Value**	**ß (SE)**	***P* Value**	**ß (SE)**	***P* Value**	**ß (SE)**	***P* Value**
Stroke type, hemorrhagic	2.51 (0.74)	<.001	0.14 (0.05)	.004	1.21 (0.2)	<.001	0.88 (0.17)	<.001	..	..	2.35 (0.81)	.004
Age	-0.16 (0.03)	<.001	-0.02 (0.002)	<.001	-0.11 (0.01)	<.001	-0.1 (0.01)	<.001	-0.01 (0.001)	<.001	-0.35 (0.03)	<.001
Sex, male	0.9 (0.67)	.18	0.17 (0.04)	<.001	0.74 (0.18)	<.001	0.47 (0.15)	.002	0.05 (0.03)	.07	2.12 (0.73)	.004
Body mass index	-0.03 (0.09)	.73	-0.01 (0.01)	.44	0.02 (0.02)	<.001	0.02 (0.02)	.25	-0.002 (0.004)	.58	0.03 (0.09)	.72
Smoking, current	0.02 (0.68)	.97	-0.002 (0.04)	.96	-0.1 (0.19)	.47	0.09 (0.15)	.57	0.004 (0.03)	.90	-0.08 (0.74)	.91
Alcohol, current	0.82 (0.62)	.19	0.04 (0.04)	.30	0.12 (0.17)	.59	0.13 (0.14)	.37	0.04 (0.03)	.17	0.93 (0.68)	.17
Education years	0.19 (0.07)	.007	0.01 (0.004)	.003	0.22 (0.02)	.47	0.24 (0.02)	<.001	0.003 (0.003)	.26	0.21 (0.08)	.006
Medical history												
Hypertension	-0.27 (0.57)	.63	-0.02 (0.04)	.67	-0.24 (0.16)	.13	-0.02 (0.13)	.88	0.003 (0.02)	.91	-0.22 (0.62)	.72
Diabetes mellitus	-1.75 (0.68)	.01	-0.16 (0.04)	<.001	-0.24 (0.19)	.20	-0.24 (0.16)	.12		..	-1.82 (0.75)	.02
Atrial fibrillation	-2.14 (1.09)	.05	-0.12 (0.07)	.08	-0.93 (0.3)	.002	-0.61 (0.25)	.01*	-0.1 (0.05)	.03	-1.69 (1.19)	.16
Hyperlipidemia		..		..		..	0.1 (0.21)	.63		..	..	..
CCAS	0.41 (0.2)	.04	0.07 (0.01)	<.001	0.22 (0.06)	<.001	0.23 (0.05)	<.001	0.02 (0.01)	.005*	1.13 (0.22)	<.001
Premorbid mRS	0.13 (0.21)	.54	-0.004 (0.01)	.79		..		..		..	-0.18 (0.23)	.43
7-day NIHSS	-0.08 (0.12)	<.001	-0.06 (0.01)	<.001	-0.09 (0.03)	.01*	-0.09 (0.03)	.001	-0.03 (0.01)	<.001	-1.07 (0.14)	<.001
Functional levels at day 7												
FMA	0.28 (0.02)	<.001	0.01 (0.001)	<.001	0.003 (0.005)	.46	0.01 (0.004)	.04	-0.0003 (0.001)	.68	0.10 (0.02)	<.001
K-MMSE	0.16 (0.07)	.03	0.02 (0.005)	<.001	0.24 (0.02)	<.001	0.14 (0.02)	<.001	0.01 (0.003)	<.001	0.38 (0.08)	<.001
FAC	0.42 (0.21)	.048	0.04 (0.01)	.004	0.07 (0.06)	.23	0.02 (0.05)	64.00	0.01 (0.01)	.11	0.60 (0.23)	.009
AHSA-NOMS	0.4 (0.23)	.08	0.002 (0.01)	.92	-0.11 (0.06)	.09	-0.05 (0.05)	.30	0.02 (0.01)	.03	0.33 (0.25)	.18
Short K-FAST	-0.09 (0.09)	.32	-0.002 (0.006)	.70	0.07 (0.02)	.008	0.12 (0.02)	<.001	-0.01 (0.004)	.008	-0.08 (0.10)	.39

Abbreviations: SE, Standard Error; FMA, Fugl-Meyer Assessment; FAC, Functional Ambulatory Category; K-MMSE, Korean Mini-Mental State Examination; Short K-FAST, Short Korean version of the Frenchay Aphasia Screening Test; AHSA-NOMS, American Speech-Language-Hearing Association National Outcome Measurement System Swallowing Scale; K-MBI, Korean modified Barthel Index; CCAS, combined condition- and age-related score in the Charlson comorbidity index; mRS, modified Rankin scale; NIHSS, National Institutes of Health Stroke Scale.

© 2022 Shin S et al. *JAMA Network Open.*
